# Management of radiation-induced oral mucositis in head and neck cancer patients: a real-life survey among 25 Italian radiation oncology centers

**DOI:** 10.1007/s00520-023-08185-5

**Published:** 2023-12-19

**Authors:** Luca Bergamaschi, Maria Giulia Vincini, Mattia Zaffaroni, Matteo Pepa, Ilaria Angelicone, Antonio Astone, Cristiana Bergamini, Sergio Buonopane, Mario Conte, Nicola De Rosa, Chiara Deantoni, Italo Dell’Oca, Davide Di Gennaro, Nadia Di Muzio, Mattia Falchetto Osti, Manuela Federico, Gianluca Ferini, Ciro Franzese, Marco Gatti, Antonietta Grillo, Vincenzo Iorio, Roberto Manzo, Luca Marmiroli, Giuseppe Martin, Federica Mazzuca, Maria Angela Molinaro, Matteo Muto, Roberto Pacelli, Alfonsina Pepe, Annarita Perillo, Donatella Russo, Francesca Salerno, Pietro Spadaro, Anna Viola, Giuseppe Carlo Iorio, Paolo Muto, Umberto Ricardi, Daniela Alterio

**Affiliations:** 1 Division of Radiation Oncology, European Institute of Oncology IRCCS, 20141 Milan, Italy; 2 Radiotherapy Department, Sant’Andrea Hospital, Sapienza University of Rome, Rome, Italy; 3 Division of Medical Oncology, Fatebenefratelli San Pietro Hospital, 00189 Rome, Italy; 4 Head and Neck Medical Oncology Unit, Fondazione IRCCS Istituto Nazionale dei Tumori, Milan, Italy; 5 Radiation Oncology Unit, Istituto Nazionale Tumori, IRCCS Fondazione G. Pascale, Naples, Italy; 6 Fondazione Muto Onlus, Casavatore, Naples, Italy; 7 Centro Aktis Diagnostica e Terapia, Marano, Naples, Italy; 8 Radiotherapy Department, IRCCS San Raffaele Scientific Institute, Vita Salute S. Raffaele University, Milan, Italy; 9 Azienda Ospedaliera Universitaria Ruggi D’Aragona, Salerno, Italy; 10 Casa di cura Macchiarella, U.O. Radioterapia Oncologica, Palermo, Italy; 11 Radiation Oncology Unit, REM Radioterapia, Viagrande, Italy; 12 Department of Radiotherapy and Radiosurgery, Humanitas Clinical and Research Center-IRCCS, Rozzano, Milan, Italy; 13 Department of Biomedical Sciences, Humanitas University, Pieve Emanuele, Milan, Italy; 14 Radiotherapy Unit, Candiolo Cancer Institute, FPO-IRCCS, Torino, Italy; 15 Radiation Oncology Unit, Azienda Ospedaliero-Universitaria Policlinico, Bari, Italy; 16 U.O. Radioterapia, Ospedale Fatebenefratelli S. Giovanni Calibita, Isola Tiberina, 00186 Rome, Italy; 17 Centro Radiologico Vega, Caserta, Italy; 18 U.O. Radioterapia A.O. Pugliese Ciaccio di Catanzaro, Catanzaro, Italy; 19 Department of Onco-Hematological Diseases, U.O.C. Radiotherapy-Azienda Ospedaliera San Giuseppe Moscati-(AV), 83100 Avellino, Italy; 20 Department of Advanced Biomedical Sciences, Federico II University School of Medicine, Naples, Italy; 21 Radioterapia Emicenter, Naples, Italy; 22 Department of Radiation Oncology, San Pio Hospital, Benevento, Italia; 23 Radiotherapy Unit, Ospedale “Vito Fazzi,”, Lecce, Italy; 24 UOC Radioterapia, ASL Roma1, Borgo S. Spirito, Rome, Italy; 25 U.O. di Oncologia ed Ematologia, Casa di Cura Villa Salus, Messina, Italy; 26 Fondazione IOM, Viagrande, Catania, Italy; 27 Department of Oncology, Radiation Oncology, University of Turin, Turin, Italy

**Keywords:** Radiation-induced oral mucositis, Head and neck cancer, Real-life survey

## Abstract

**Aim:**

Radiation-induced oral mucositis (RIOM) is the most frequent side effect in head and neck cancer (HNC) patients treated with curative radiotherapy (RT). A standardized strategy for preventing and treating RIOM has not been defined. Aim of this study was to perform a real-life survey on RIOM management among Italian RT centers.

**Methods:**

A 40-question survey was administered to 25 radiation oncologists working in 25 different RT centers across Italy.

**Results:**

A total of 1554 HNC patients have been treated in the participating centers in 2021, the majority (median across the centers 91%) with curative intent. Median treatment time was 41 days, with a mean percentage of interruption due to toxicity of 14.5%. Eighty percent of responders provide written oral cavity hygiene recommendations. Regarding RIOM prevention, sodium bicarbonate mouthwashes, oral mucosa barrier agents, and hyaluronic acid-based mouthwashes were the most frequent topic agents used. Regarding RIOM treatment, 14 (56%) centers relied on literature evidence, while internal guidelines were available in 13 centers (44%). Grade (G)1 mucositis is mostly treated with sodium bicarbonate mouthwashes, oral mucosa barrier agents, and steroids, while hyaluronic acid-based agents, local anesthetics, and benzydamine were the most used in mucositis G2/G3. Steroids, painkillers, and anti-inflammatory drugs were the most frequent systemic agents used independently from the RIOM severity.

**Conclusion:**

Great variety of strategies exist among Italian centers in RIOM management for HNC patients. Whether different strategies could impact patients’ compliance and overall treatment time of the radiation course is still unclear and needs further investigation.

**Supplementary Information:**

The online version contains supplementary material available at 10.1007/s00520-023-08185-5.

## Introduction

Head and neck cancer (HNC) represents the seventh most common cancer worldwide [1]. They account for different histologies (mainly squamous cell carcinoma but also salivary glands tumors, undifferentiated carcinoma, melanoma, lymphomas…) located in different head and neck subsites (oral cavity, pharyngeal axis, larynx, paranasal sinuses, and salivary glands).

Radiotherapy (RT) is a cornerstone treatment for cancers of the head and neck region [2]. It is indicated either as an exclusive treatment or in patients at high risk of local recurrence after surgery. The total dose of RT ranges from 45 to 70 Gy, administered mainly using a standard fractionation schedule (1.8–2.2 Gy/fraction, 1 fraction/day for 5 fractions/week). Platinum-based concurrent chemotherapy is indicated in patients with locally advanced stage tumors (stage III or IV according to NCCN guidelines) in the presence of pathological features such as positive surgical margins and/or extracapsular extension in the postoperative setting [3].

Radiation-induced oral mucositis (RIOM) is the most frequent and dose-limiting radiation-related side effect in the setting of patients treated with curative RT for HNC [4, 5]. Pain and severe dysphagia due to RIOM may lead to significant patient weight loss with an overall worsening of his/her performance status. Most importantly, RIOM often causes the temporary interruption of the radiation course which has been demonstrated to decrease the efficacy of the radiation treatment [6, 7].

Different scales for grading the severity of RIOM are currently used in clinical practice, as a guide for the radiation oncologist in undertaking preventive and therapeutic strategies [8]. For instance, the Radiation Therapy Oncology Group (RTOG)/European Organisation for Research and Treatment of Cancer scale considers either the anatomical changes of the oral mucosa (from grade 0 with no toxicity to grade 4 mucosal ulceration/hemorrhage and necrosis) or the level of pain reported by patients caused by RT [9]. Instead, the Common Terminology Criteria Adverse Event (CTCAE V5.0, November 27, 2017) scale distinguishes different cases, from asymptomatic ones (grade 1), which do not require any medical intervention, to more serious cases requiring urgent nutritional and/or medical intervention (grade 4) or leading to the death of the patient (grade 5).

Despite its clinical relevance, a standardized strategy for preventing and treating RIOM has not been defined yet [10–14]. Recommendations provided by cooperative group of experts have been published to guide the management of RIOM in daily clinical practice [15–17]. Nevertheless, to date, strategies applied to manage RIOM remain at institutional and/or personal levels according to internal guidelines and professional’s expertise.

Aim of the present work is to perform a real-life survey on how RIOM is managed among Italian radiation therapy centers. Moreover, whether the volume of treated patients could have an impact on the single-institution strategy has been also analyzed.

## Materials and methods

On 11 May 2022, an online survey composed of a total of 53 questions, including both multiple-choice and open-ended ones, was administered through a personnel contact to radiation oncologists working in 25 different RT centers across Italy.

The survey was composed of 40 questions divided into three sections: (i) retrospective analysis of patients with HNC treated in 2021 in each center; (ii) strategies generally used for the prevention of RIOM; and (iii) strategies used for the treatment of RIOM in daily clinical practice. The full text of the survey is available in the supplementary materials section (supplementary material S1). All the participants gave their consent to the publication and the use of collected data for scientific purposes.

In the retrospective analysis, oncologic treatment characteristics (in terms of radiation technique and concurrent systemic treatments), overall RT treatment time, and treatment interruptions were collected.

Among general strategies applied to prevent and treat RIOM, data on institutional organization (professionals who manage RIOM, availability of dedicated nurses, and/or access to supportive care, nutrition, speech, and psychological services) and use of a standardized approach (RIOM data collection using validated scales and adherence to internal and/or published guidelines) has been also collected. The approach (prophylactic or therapeutic intervention) to artificial nutrition (both enteral and parenteral) was investigated.

All agents used in daily clinical practice both in the prevention and treatment phase of RIOM have been collected and gathered (when feasible).

Data will be presented as mean/median across the responders (section i) and counts (sections ii and iii). Moreover, an arbitrary cut-off of 50 treated patients/year has been used to define centers as “high-volume” (>50 patients/year) or “low-volume” (< 50 patients/years) centers. Results were divided accordingly to compare the two groups in terms of treatment strategies.

## Results

All the 25 contacted RT centers responded to the survey and all sections have been completed. Dividing according to geographical location, seven centers are located in northern Italy, five in central Italy, and the remaining in southern Italy.

## Section 1: questionnaire

### Retrospective analysis

In 2021, a total of 1554 patients with HNC were treated in the 25 centers participating (median 54, IQR: 20–70). The majority (median 91%) of the treatments had a curative intent (36% of them postoperative), while the others were administered for palliative intent. In most cases (mean 84%), patients underwent intensity-modulated RT (IMRT) technique. One center used a 3D RT technique for all patients, while the remaining 24 centers applied this technique on a median of 17% of patients.

Platinum-based chemotherapy was the most (71% of patients) frequently concurrent treatment, with a median of 43% and 29% of patients treated using a weekly and 3-weekly schedule, respectively. Cetuximab was used in 17 centers to treat a mean of 10% of patients.

The median value of median overall treatment time was 41 days (IQR: 35–45). The mean percentage of patients who interrupted treatment due to RT-related toxicity was 14.5% (data available for 19 centers). A median of 6% (IQR: 3.5–15) of patients required enteral nutrition.

### Strategies for RIOM prevention

In almost all centers (96%), HNC patients are visited at least once a week during the RT course. Quality of life questionnaires were distributed to the patients in 16% of centers and the collection of pain data on a quantitative scale is provided in 80% of cases. In-treatment toxicity is collected systematically at least once a week (84% of facilities), using the CTCAE scale (38%), the RTOG scale (19%), or both (43%). In the case of concurrent chemotherapy, enteral nutrition is proposed only to patients with significant weight loss during the RT course and to all fragile patients in 48% and 20% of cases, respectively (20% in both situations). All but three centers (which never use parenteral nutrition) used similar criteria to select patients candidates for parenteral nutrition.

The majority of the centers (80%) provide patients with written oral cavity hygiene recommendations. Among 17 centers (data not available for three centers), accurate daily oral cavity cleaning (52%), use of mucosal barrier agents (47%), and pre-treatment dentistry evaluation (35%) were the most frequent recommendations.

About half of the centers (52%) use internal guidelines for RIOM prevention, and 15 centers referred to literature evidence and/or expert recommendations (60% of recommendations provided by the Associazione Italiana di Radioterapia ed Oncologia Clinica — AIRO). Eighteen (72%) centers provided written general recommendations for RIOM prevention. Data from 14 centers (data not available for four centers) accurate oral cavity cleaning (60%), oral mouthwashes with bicarbonate (47%), and pre-treatment dentistry evaluation (40%) were the most frequent pieces of advice.

**Fig. 1:**
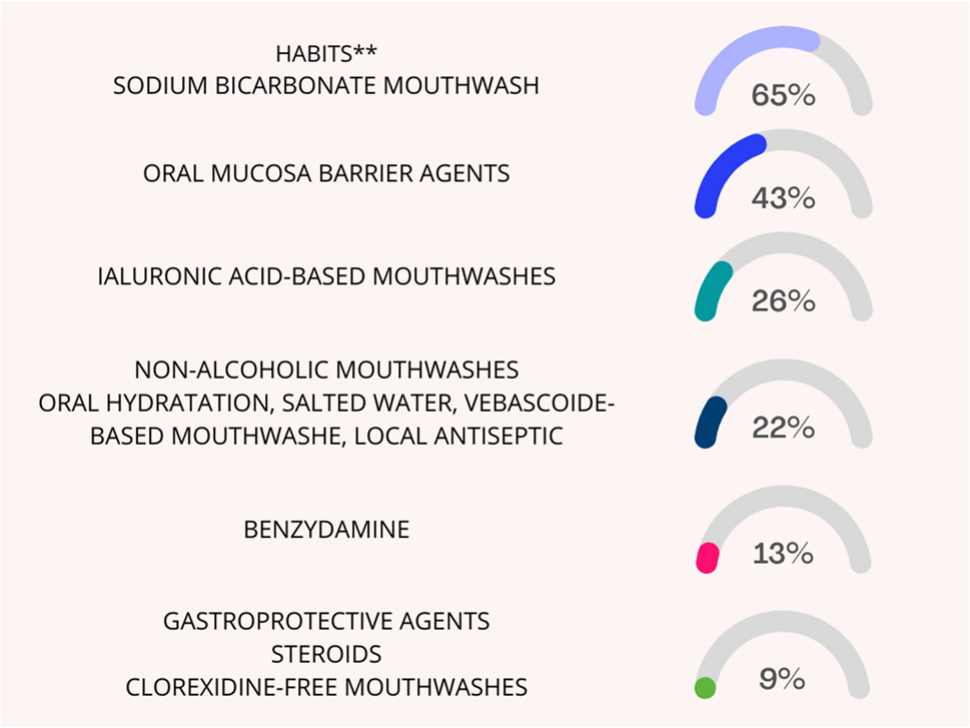
Summary of the products and agents for the prevention of oral mucositis (left) and the percentage of centers in which they are indicated (right)*. Percentages are calculated on 23 centers that reported using prevention products

All but four centers also suggest the use of topic and/or systemic agents to prevent RIOM (Fig. 1).

Six centers produce galenic products (a mixture of different agents) produced by their pharmacy.

### Strategies for RIOM treatment

The radiation oncologist manages the acute RIOM toxicity in all but one center: medical oncologists and pain specialists also support patient care in 12 (48%) and eight centers, respectively. In 16 (64%) centers, hospitalization for supportive care is allowed. Moreover, different services contribute to taking care of patients during the radiation treatment course: 13 (52%) centers have a nurse dedicated to HNC patients, 18 (72%) centers have a supportive therapy service for pain management, 22 (88%) centers have nutritional consultants, 14 (56%) centers have a speech therapy service for the management of mechanical dysphagia while 18 (72%) have a psycho-oncology service as well.

Fourteen (56%) centers base treatments of RIOM on literature evidence, while internal guidelines are present in 13 centers (44%). Eight centers (32%) did not follow either internal guidelines or literature data. Galenic agents were produced by pharmacies of seven institutions.

**Fig. 2:**
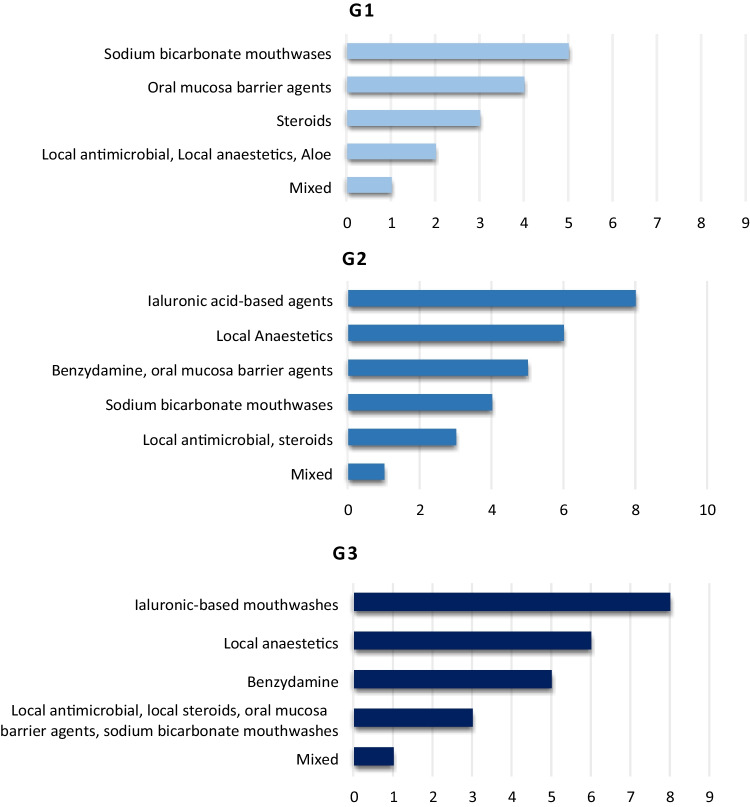
Topic agents for mucositis grades 1, 2, and 3

**Fig. 3:**
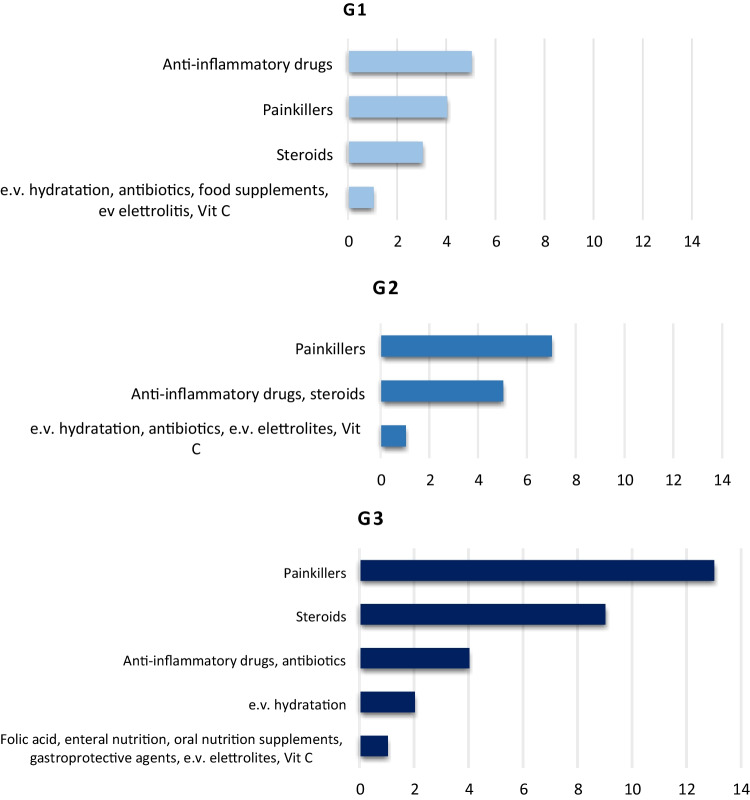
Systemic agents for mucositis grades 1, 2, and 3

The frequency of topic and systemic agents used to treat RIOM is reported in Figs. 2 and 3, respectively.

**Fig. 4:**
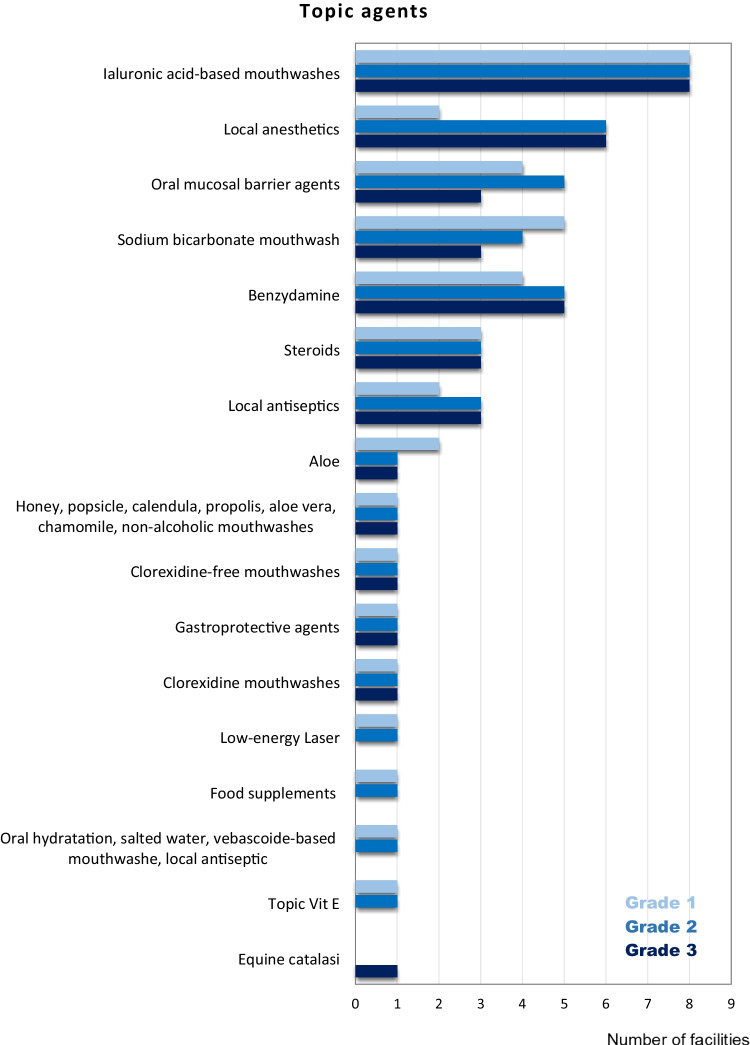
Summary of the topical agents used for the treatment of radiation-induced oral mucositis

**Fig. 5:**
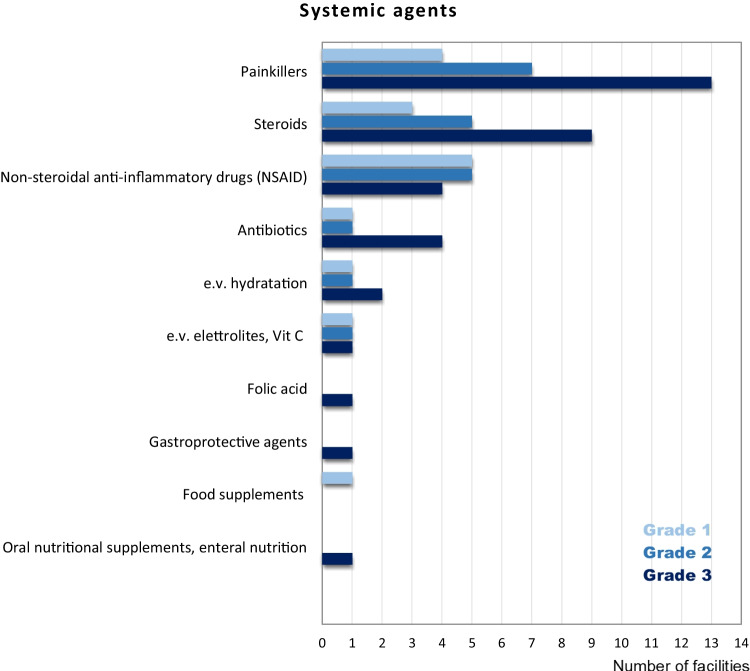
Summary of the systemic agents used for the treatment of radiation-induced oral mucositis

The frequency of topic and systemic agents used to treat RIOM according to the increasing grade of mucositis (from G1 to G3) is reported in Fig. 4 and Fig. 5, respectively.

### Volume of treated patients

To investigate the impact of the “volume of patients” (number of patients treated per year) on the management of RIOM, we classified centers as “low-volume” (< 50 patients/years) and “high-volume” (> 50 patients/years). Twelve and 13 centers gathered low- and high-volume cohorts, respectively. The mean number of patients treated in each group was 21 (IQR: 10–25) and 96 (IQR: 66–136) for low- and high-volume centers, respectively.

Differences in terms of RT technique, concurrent systemic agents, center supportive network, and prevention/treatment strategies between high- and low-volume centers are reported in Table 1.

**Table 1:** Results from the questionnaire dividing centers into low- and high-volume facilities

		Low-volume facilities (*n* = 12)	High-volume facilities (*n* = 13)
Radiotherapy	IMRT (mean % of treated patients)	80%	94%
Chemotherapy	Weekly CDDP (mean % of treated patients)	69%	37%
	Three-weekly CDDP (mean % of treated patients)	17%	49%
	Cetuximab	11%	9%
Patients requiring enteral nutrition (mean)		14%	12%
Quality of life questionnaire collection		8%	23%
Use of published guidelines/recommendation to prevent RIOM		58%	61%
Use of published guidelines/recommendation to treat RIOM		50%	61%
Availability of a dedicated nurse		42%	53%
Availability of supportive care service for pain		67%	77%
Availability of nutrition service		91%	85%
Availability of speech service		50%	62%
Availability of psycho-oncology service		83%	62%
Availability of patients hospitalization		61%	66%

The mean value of the overall treatment time resulted to be 42 (IQR: 38–45) and 40 (IQR: 30–45) days in low- and high-volume centers, respectively. The percentage of patients who interrupted the RT treatment according to low- and high-volume classification was 16% and 13%, respectively.

## Discussion

Results of the present survey confirmed that a great variety exists among Italian centers in the management (prevention and treatment) of RIOM in the setting of HNC. Of note, the majority of participating centers are provided with different supportive care services and follow internal guidelines and/or literature recommendations. Moreover, a low number of patients (<15%) interrupted the RT treatment course and the mean overall treatment time (41 days) remains quite low.

Despite a large amount of literature data, few agents reached level 1 evidence (results coming from prospective randomized trials and/or meta-analysis) for the management of RIOM. In this scenario, recommendations derived from the consensus of experts and literature review have been published over the last decades. Since 2004, the Multinational Association of Supportive Care in Cancer and the International Society of Oral Oncology (MASCC/ISOO) cooperative group published their recommendations on the prevention and treatment of RIOM [18–21]. Similarly, the European Society of Medical Oncology (ESMO) has periodically published its recommendation since 2009 [15, 22], while an Italian working group endorsed by Associazione Italiana di Radioterapia ed Oncologia Clinica (Gruppo AIRO Inter-regionale Lazio-Abruzzo-Molise) did it in 2019 [17].

### Prevention of RIOM

Results of MASCC/ISOO, ESMO, and AIRO recommendations on the prevention of RIOM are summarized in Table 2.

**Table 2:** Results of MASCC/ISOO, ESMO, and AIRO recommendations on the prevention of radiation-induced oral mucositis

Agents	MASCC/ISOO 2020 Level of evidence	ESMO 2015 recommendation	AIRO 2018 recommendation	n. of centers
Accurate oral hygene	**Recommended** Expert opinion	**Recommended** Expert opinion	**Recommended**	16
Pre-treatment Dental evaluation	**Recommended** Expert opinion	**Recommended** Expert opinion	**Recommended**	7
Saline solution	**Recommended** Expert opinion	**Not recommended**	**Recommended**	1
Sodium bicarbonate	**Recommended** Expert opinion	**Recommended** Expert opinion	**Recommended**	15
Benzydamine mouthwash	**Recommended** RT < 50 Gy; level I **Suggested** CRT; level II	**Recommended** RT Level I	**Recommended** RT moderate doses without CT	3
Low-energy laser	**Recommended** RT Level II **Recommended** CRT Level I	**Suggested** Level III	**Recommended**	1
Oral glutamine	**Suggested CRT** Level II	NR	**Not recommended**	1
Honey	**Suggested** Level II	NR	NR	0
Zinc supplements	NR	**Suggested** Level III	NR	
Mucoadhesive solution	NR	NR	**Not recommended**	10
Chlorhexidine	**Not recommended**	**Not recommended**	**NR**	1
Sucralfate	**Not recommended**	**Not recommended**	**Not recommended**	3
Antibiotics	**NR**	**Not recommended**	**Not recommended**	0
Granulocyte growth factors	**Not recommended**	**Not recommended**	**Not recommended**	0
Antimicrobial lozenge	**Not recommended**	**Not recommended**	**Not recommended**	0
Misoprostol mouthwash	**NR**	**Not recommended**	**NR**	0
Systemic pilocarpine	**NR**	**Not recommended**	**NR**	0

*CTRT* chemoradiation, *NA* not applicable, *NR* not reported

Although supported by low-level evidence of literature, pre-treatment dental evaluation, accurate oral hygiene, and sodium bicarbonate were recommended as standard of care for all adult patients candidates to RT for HNC [15–17]. Among the participating centers, two centers do not provide any recommendations to prevent RIOM. Of the remaining 19 (four centers used internal guidelines and details were not provided), sodium bicarbonate was the most frequently used agent (70% of the centers). Data on the use of saline solution were more controversial and were not considered robust enough by the ESMO panelists. In the present survey, only one center advised patients to use saline solution mouthwashes to prevent RIOM.

Benzydamine and low-energy laser (LEL) were recommended for the prevention of RIOM both in patients treated with RT alone and in those treated with chemoradiation [15]. Benzydamine is a non-steroidal anti-inflammatory drug with anesthetic, analgesic, and antiseptic properties. A randomized multicentric randomized double-blind placebo-controlled trial demonstrated the efficacy of benzydamine for RIOM prevention [23]. A total of 172 subjects (84 treated with benzydamine and 88 with placebo) were enrolled in 16 North American centers. Benzydamine oral rinse (1.5 mg/ml) or placebo was administered before, during RT, and for 2 weeks from the end of treatment. Results showed that benzydamine produced a 26.3% reduction of mucositis in area under curve compared to placebo (*p* = 0.009). In particular, benzydamine produces a statistically significant benefit at high RT doses (range 25–37.5 Gy, *p* < 0.001, and range 37.5–50 Gy, *p* = 0.006) while it was not effective in patients treated with a slight hypofractionated schedule (>2.2 Gy/fraction). Moreover, 33% of patients treated with benzydamine remained free from ulcers compared to 18% of the placebo group (*p* = 0.037). Subsequently, four more prospective studies confirmed the efficacy of benzydamine in preventing and reducing the severity of oral mucositis in patients treated with RT [24–27]. Despite literature evidence and recommendations, only three (12%) centers participating in the present survey stated to have benzydamine in their armamentarium to prevent RIOM.

LEL stimulates the biological responses to repair injuries in healthy tissues and is therefore included among the photobiomodulation therapies. A double-blind randomized trial (low-energy He-Ne laser vs placebo-light treatment) has been published in 1999 by Bensadoun et al. [28]. Thirty patients were enrolled and received a daily application with laser/placebo during the whole course of RT. Results showed that the mean grade of mucositis was significantly lower in patients treated with LEL compared to the control group (grade 1.7 ± 0.26 vs 2.1 ± 0.26, respectively, *p* = 0.01) with the highest differences observed during the last weeks (from 4th to 7th) of treatment. Moreover, the preventive use of the laser also allowed a significant reduction in oral pain (*p* = 0.025). Subsequent studies confirmed the efficacy and safety of LEL in adult patients with HNC treated with RT [29–31]. A recent position paper published by the World Association of photobiomoduLation Therapy stated that literature evidence is robust enough for the clinical application of LEL to prevent oral mucositis as well as in other settings of treatment-induced toxicities [32]. The reported data led to the inclusion of LEL in all published recommendations and guidelines. Despite this, only one center involved in the present survey uses LEL in its clinical practice. On the contrary, some other products like mucoadhesive agents, chlorhexidine, and sucralfate are used by 10, 1, and 3 centers respectively although not supported by robust data. Of note, oral mucosa barrier and hyaluronic acid-based agents are routinely used by 43 and 26% of centers, respectively, despite not being mentioned within the above-cited published recommendations.

### Treatment of RIOM

Results of MASCC/ISOO, ESMO, and AIRO recommendations on the treatment of RIOM are summarized in Table 3.

**Table 3:** Results of MASCC/ISOO, ESMO, and AIRO recommendations on the treatment of radiation-induced oral mucositis

Agents	MASCC/ISOO 2020 Recommendation Level of evidence	ESMO 2015 Recommendation Level of evidence	AIRO 2018 Recommendation	Number of centers		
				G1	G2	G3
Saline solution	**Recommended** Expert opinion	**Not recommended**	**Recommended**	1	1	0
Sodium bicarbonate	**Recommended** Expert opinion	**Recommended** Expert opinion	**Recommended**	5	4	3
Low-energy laser	NR	NR	NR	1	1	0
Local anestetics (topical morphine or lidocanine)	Morphine **Suggested** Level III	Morphine **Suggested** Level III	Morphine recommended	2	6	6
Doxepin mouthwash	NR	**Suggested** Level IV	NR	0	0	0
Mucoadhesive solution	**Not recommended**	NR	**Recommended**	4	5	3
Chlorhexidine	NR	**Not recommended**		1	1	1
Sucralfate	**Not recommended**	**Not recommended**	**Not recommended**	1	1	1
Antibiotics	NR	NR	**Not recommended**	1	1	4
Antimicrobic lozenge	NR	**Not recommended**	NR	2	3	3

With regard to strategies aiming to treat RIOM, only topical morphine resulted to be indicated to reduce oral pain. Its use is suggested by either MASCC/ISOO or ESMO recommendations. In a double-blind study, Sarvizadeh et al. randomized 30 patients to treat grade 3 mucositis with topical morphine or a magic mouthwash (magnesium aluminum, hydroxide, lidocaine, and diphenhydramine) for a period of 6 days. On the last day of treatment, mucositis resulted significantly lower in the study group compared to the control cohort (*p* = 0.045) [33]. Similar results were obtained in a group of 26 patients randomly assigned to receive topical morphine or magic mouthwashes [34]. The duration of severe pain as well as pain intensity resulted lower in patients who received morphine compared to the control group. In the present survey, only one center prescribes topical morphine to treat any grade of mucositis, while other agents like mucoadhesive solution and chlorhexidine are more frequently used.

Hyaluronic acid-based agents are the most frequent products administered by respondents to the survey. A recent meta-analysis showed that it was beneficial for both cutaneous and mucosal radiation-induced side effects (RR: 0.14, 95% CI: 0.04 to 0.45) [35].

### Overall treatment time

The occurrence of RIOM causes pain and dysphagia which produce patients’ weight loss and worsening of overall treatment compliance. This aspect may lead to interruptions of the RT course due to uncontrolled side effects. Worsening of oncological outcomes occurs in patients who did not complete the RT treatment course within the planned time. González Ferreira et al. [7] carried out a literature review and showed that delays in RT could produce an average loss of locoregional control ranging from 1.2% per day to 12–14% per week of interruption [7]. Moreover, it has been estimated that a daily dose increase of about 0.6–0.8 Gy would be required to compensate for each day of overall treatment time prolongation. The median value of the overall treatment time (considering both curative and postoperative treatments) reported by centers participating in the present survey resulted to be quite low (41 days, IQR: 35–45). Based on this finding, two main considerations can be done: (1) despite the wide variety of approaches both in preventing and treating RIOM, their impact on the overall treatment course seems to be low and (2) a network of supportive care services (including management of pain, nutrition, and psychological support as well as hospitalization if required) is provided by the majority of RT facilities and this aspect could have done a positive impact on patients’ compliance.

### Volume of treated patients

The definition of “high” and “low” volume centers (in terms of hospital volume and/or professional experience) has not been established yet. Nevertheless, it has been demonstrated that the higher the number of patients treated, the better the oncological outcomes. Eskander et al. performed a systematic review of the literature and showed that high-volume hospitals (HR 0.886, 95% CI 0.820–0.956) achieved better results in terms of patients’ long-term survival [36]. In order to quantify the impact of the centers’ experience on the management of RIOM, we performed a subgroup analysis according to the number of treated patients/year using an arbitrary cut-off of 50 patients. Results showed that in high-volume centers, modern RT (namely IMRT) and standard high-dose chemotherapy (3-weekly CDDP) are more frequently used compared to low-volume centers. Similarly, the overall use of published RIOM-related recommendations and availability of supportive care services are slightly higher in high-volume centers for the majority of the considered parameters. Nevertheless, the mean overall treatment time resulted similar between the two groups as well as the number of patients who required a treatment interruption due to RIOM-related toxicity.

### Limitations

Several criticisms burdened the present study. Centers participating in the present survey represent only 15% of Italian RT facilities [37]. Nevertheless, the spread of geographical distribution among south (28%), center (20%), and northern Italy (52%), as well as the variability of treated patients/year number (centers at high and low volume), ensure that reported results could be considered representative of how RIOM is nowadays managed among Italian centers in the daily clinical practice. Moreover, several other drugs and agents reported in the literature (i.e., anti-oxidant or immunonutrition) have not been considered in the present analysis. Finally, other factors than mucositis (such as patient’s age, comorbidities, treatment characteristics, and dysgeusia) could impact the patient’s compliance with the radiation treatment.

## Conclusions

To the best of our knowledge, this is the first study reporting an accurate snapshot of the Italian attitude on which agents and drugs are currently used in daily clinical practice to prevent and treat RIOM in Italian RT facilities. Results showed that a great variety still exist despite the availability of national and international recommendations. In this scenario, whether different strategies to manage RIOM could impact patients’ compliance and overall treatment time of the radiation course is still unclear and requires further investigation. Moreover, the presented findings strongly encourage efforts to standardize the RIOM management protocols in daily clinical practice among the RT facilities. To this aim, similar analyses in other countries would be useful to highlight eventual geographical differences.

### Supplementary information

ESM 1 (DOCX 16 kb)

## Abbreviations

AUC: area under curve
HNC: head and neck cancer
IMRT: intensity-modulated radiotherapy
NSAID: non-steroidal anti-inflammatory drug
LEL: low-energy laser
RIOM: radiation-induced oral mucositis
RT: radiotherapy
QoL: quality of life
