# Supplementary material for: Management of radiation-induced oral mucositis in head and neck cancer patients: a real-life survey among 25 Italian radiation oncology centers
Source: Support Care Cancer. 2023 Dec 19;32(1):38. doi: 10.1007/s00520-023-08185-5 (PMC10728275; doi:10.1007/s00520-023-08185-5)
Supplement: Supplementary file 1 — (DOCX 16 kb) [file 520_2023_8185_MOESM1_ESM.docx]

**Supplementary Material S1**

Questionnaire

**Section 1 – Retrospective analysis and general information**

**1.a Head and neck cancer patients treated in 2021**

1. Head and neck cancer patients treated at your center in 2021. (number)
2. Median age of patients treated in 2021 in your center.
3. Intent to treatment (% of curative/postoperative/palliative treatments)
4. Radiation techniques used (% of Intensity Modulated Radiotherapy/3D conformal Radiotherapy/Brachytherapy)
5. Concurrent systemic treatments (weekly cisplatin/ three-weekly cisplatin/ cetuximab/ others)
6. Median duration of the Radiation treatment course (days).
7. Number of patients who required enteral nutrition during the radiation course (percentage of patients)

**1.b General organization of the center**

1. Are in-treatment visits performed at least once a week? (yes/no)
2. In patients treated with concurrent chemoradiation, enteral nutrition is proposed:
   1. as a preventive approach to all fragile patients (ie elderly patients with significant comorbidities) or in case of high risk of ab-ingestis peumonitis
   2. only to patients experiencing a significant weight loss
   3. others
3. Do you collect routinely collect Quality of Life questionnaires? (yes/no)
4. Do you routinely collect the symptom of pain according to a quantitative scale? (yes/no)
5. Do you routinely collect treatment-related toxicity on a weekly basis? (yes/no)
6. If yes, which scale do you generally use (RTOG/CTCAE/WHO/others)
7. Is it possible to be hospitalized for supportive care? (yes/no)
8. Does your center provide a dedicated nurse? (yes/no)
9. Does your center provide supportive care service to manage treatment-induced pain? (yes/no)
10. Does your center provide a nutritional service? (yes/no)
11. Does your center provide a speech service? (yes/no)
12. Does your center provide a psyco-oncology service? (yes/no)
13. In your center, which professional figure generally manage RIOM (radiation oncologist/medical oncologist/others)

**Section 2 Prevention of RIOM**

1. Use of written institutional recommendations to prevent RIOM. (yes/no). If yes, to specify
2. Use of international published recommendations to prevent RIOM. (yes/no). If yes, to specify
3. Use of written general recommendations for the oral hygiene. (yes/no). If yes, to specify
4. Use of written general recommendations for prevention of RIOM. (yes/no). If yes, to specify
5. Use of topic agents or drugs to prevent RIOM (yes/no). If yes, specify
6. Use of systemic drugs to prevent RIOM (yes/no). If yes, specify
7. Use of galenic agents to prevent RIOM (yes/no). If yes, specify

**Section 3 Treatment of RIOM**

1. Use of written institutional recommendations to treat RIOM. (yes/no). If yes, specify
2. Use of international published recommendations to treat RIOM? (yes/no). If yes, specify
3. Use of galenic agents to treat RIOM (yes/no). If yes, specify
4. Which topic agents to treat Grade 1 RIOM. (open answers)
5. Which systemic agents to treat Grade 1 RIOM. (open answers)
6. Which topic agents used to treat Grade 2 RIOM. (open answers)
7. Which systemic agents to treat Grade 2 RIOM. (open answers)
8. Which topic agents to treat Grade 3 RIOM. (open answers)
9. Which systemic agents to treat Grade 3 RIOM. (open answers)

**Abbreviations:**

RIOM= Radiation-Induced Mucositis

CTCA= Common Terminology Criteria Adverse Events

RTOG= Radiation Therapy Organitazion Grade

WHO= World Health Organization
